# Rural-urban differences in gut microbiome among Vietnamese women

**DOI:** 10.64898/2026.10.04.26364707

**Published:** 2026-10-06

**Authors:** Justin Guo, Huong T.T. Tran, Qiuyin Cai, Minh V. Hoang, Thuan V. Tran, Xiao-Ou Shu, Sang M. Nguyen

## Abstract

Gut microbiome plays an important role in human health. Urbanization has been suggested to alter the gut microbiome. We comprehensively evaluated differences in gut microbiome profiles of 76 urban and 81 rural female residents of Vietnam using shotgun metagenomic data. We evaluated rural-urban differences in gut microbial diversity, individual microbial taxa, and metabolic pathways. Rural residents had higher alpha and beta diversities in age-adjusted models (p<0.05). Additionally, significant rural-urban differences were observed for 35 microbial taxa in age-adjusted models (FDR<0.1). Rural residents had higher relative abundance of classes, orders, and families of *FGB2982* and *FGB38642* and strain *SGB15332* of the species *Faecalibacterium prausnitzii* , whereas urban residents had higher relative abundances of species *Bacteroides thetaiotaomicron, Coprobacter fastidiosus, Bilophila wadsworthia, Enterobacter cloacae, Enterobacter hormaechei, Klebsiella aerogenes* , *Dielma fastidiosa,* and *Holdemania massiliensis.* Notably, these associations were attenuated after further adjustment for socioeconomic characteristics, lifestyle, dietary factors, and comorbidity (FDR>0.1). In the fully adjusted model, significant rural-urban differences were observed in the abundance of 17 microbial taxa, including *Ruminococcus sp AF13 28* and *Prevotella pectinovora,* as well as in three metabolic pathways (FDR<0.1). This study suggests that urbanization-related factors may contribute to differences in the gut microbiome between urban and rural Vietnamese women.

## Full Text Availability

The license terms selected by the author(s) for this preprint version do not permit archiving in PMC. The full text is available from the preprint server.

